# Patient-reported non-motor outcomes after endovascular thrombectomy and intravenous thrombolysis: an observational study

**DOI:** 10.1093/esj/aakag066

**Published:** 2026-06-22

**Authors:** Hatice Ozkan, Gareth Ambler, Yezen Sammaraiee, Alex P Leff, David J Werring, Robert J Simister

**Affiliations:** Stroke Research Centre, Department of Translational Neuroscience and Stroke, UCL Queen Square Institute of Neurology, London, United Kingdom; National Hospital for Neurology and Neurosurgery, University College London Hospitals NHS Foundation, Queen Square, London, United Kingdom; Department of Statistical Science, University College London, Gower Street, London, United Kingdom; Stroke Research Centre, Department of Translational Neuroscience and Stroke, UCL Queen Square Institute of Neurology, London, United Kingdom; National Hospital for Neurology and Neurosurgery, University College London Hospitals NHS Foundation, Queen Square, London, United Kingdom; Stroke Research Centre, Department of Translational Neuroscience and Stroke, UCL Queen Square Institute of Neurology, London, United Kingdom; National Hospital for Neurology and Neurosurgery, University College London Hospitals NHS Foundation, Queen Square, London, United Kingdom; Stroke Research Centre, Department of Translational Neuroscience and Stroke, UCL Queen Square Institute of Neurology, London, United Kingdom; National Hospital for Neurology and Neurosurgery, University College London Hospitals NHS Foundation, Queen Square, London, United Kingdom

**Keywords:** health-related quality of life, non-motor, patient-reported, revascularisation, stroke

## Abstract

**Introduction:**

Adverse non-motor outcomes dominate the lived reality of post-stroke recovery, yet remain poorly understood after intravenous thrombolysis (IVT), endovascular thrombectomy (EVT) or both. We characterised the prevalence of outcomes in 13 non-motor domains, stratified by mRS scores, and identified baseline factors associated with adverse outcomes at 6 months follow-up.

**Methods:**

We conducted a prospective observational sub-study within the Stroke Investigation Group in North and Central London (SIGNAL) registry to characterise non-motor outcomes after IVT, EVT or both. At 6 months, we assessed mRS alongside 13 patient-reported non-motor domains, including neuropsychiatric, fatigue, sleep, social participation, sensory, autonomic and cognitive outcomes. We used unadjusted analysis to estimate prevalence and adjusted multivariate logistic regression to investigate associated baseline factors.

**Results:**

We included 642/646 (99.3%) eligible surviving patients (median age 73 years; 43.5% female; median NIHSS = 5; mRS = 1) treated with IVT, EVT or both. At 6 months, the prevalence of adverse non-motor outcomes across 13 domains ranged from 18% to 56% across all treatment groups. Among patients with a favourable functional outcome (mRS 0–2, *n* = 409), fatigue (51.3%), sleep disturbance (47.6%) and mood problems (39.6%) were most prevalent. In those with an unfavourable outcome (mRS 3–5, *n* = 233), dependency in activities of daily living (51.0%), reduced social participation (44.3%) and bladder dysfunction (41.0%) were common. Stroke recurrence, female sex and baseline NIHSS > 5 were significantly associated with multiple adverse non-motor outcomes at 6 months.

**Conclusion:**

Despite favourable mRS, a high proportion of patients treated with IVT and/or EVT report adverse non-motor outcomes. Systematic non-motor assessments alongside mRS are needed to accurately capture post-stroke symptom burden and guide person-centred life after stroke care.

## Graphical Abstract

**Graphical Abstract:**
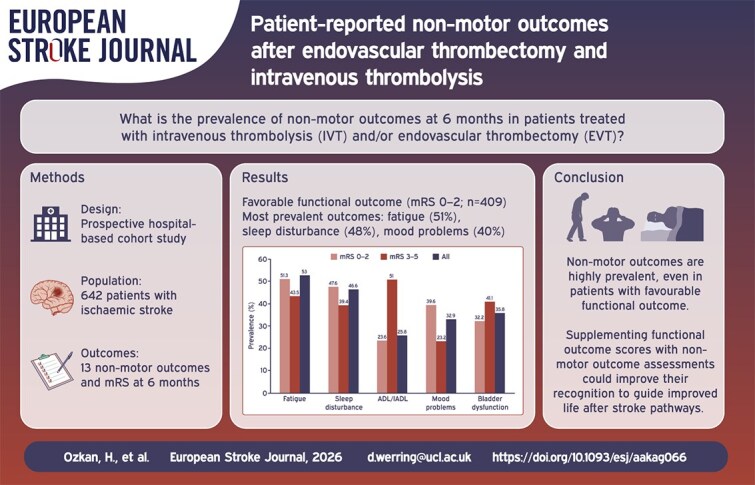

## Introduction

Stroke causes a profound global health burden. In 2021, ischaemic stroke accounted for 65.3% of all stroke events, affecting 7.8 million people and resulting in 70.4 million disability-adjusted life years lost.^1^ Time-critical reperfusion therapies have transformed acute ischaemic stroke care over the past 2 decades; intravenous thrombolysis (IVT), and EVT have become standard practice for eligible patients, substantially improving survival and reducing disability.^2–6^

Functional outcome after reperfusion is conventionally assessed using the mRS, which remains the dominant metric in clinical trials and practice.^2–6^ However, the mRS is a global measure of disability that lacks sufficient detail to detect changes within non-motor outcome domains, such as neuropsychiatric, sensory, autonomic and cognitive function.^7^ Specific adverse non-motor outcomes after stroke include anxiety, depression, fatigue, sleep disturbance, social participation, pain, bowel dysfunction, bladder dysfunction, mood problems, communication problems, reduced activities of daily living (ADL), memory and thinking problems.^8^^,^^9^ These challenges represent the lived reality of life after stroke, with substantial consequences for survivors and their caregivers, as well as major socioeconomic implications, yet they are often reported only as post-hoc or supplementary analyses in major ischaemic stroke revascularisation trials.^10^^,^^11^

Emerging post-hoc data suggest that the prevalence of adverse non-motor outcomes after reperfusion treatments might be substantial, even among patients with relatively favourable mRS scores.^10–12^ In a multicentre cohort of 515 patients treated with EVT, 43.9% reported anxiety or depression at 90 days despite a median mRS of 2; in another cohort of 504 patients treated with EVT, 40%–42% reported pain, 50%–66% had ADL impairments and more than 50% experienced fatigue, with a median mRS of 1.^10^^,^^11^ Moreover, in a cohort of 165 patients treated with EVT, the reported prevalence was 30% for reduced ADL, 35% for pain and 38% for anxiety or depression.^12^ Previously reported risk factors for these outcomes include older age, higher baseline stroke severity and pre-stroke disability.^10–12^ These previous findings suggest that non-motor outcomes, such anxiety, depression, pain and dependency with ADL are common even among patients with relatively favourable outcomes.^10–12^ While an increase in raw mRS scores may capture certain non-motor domains, including memory problems, dependency in ADLs and autonomic dysfunctions such as bowel or bladder dysfunctions, the lack of granularity in the commonly used binary classification of mRS limits its ability to fully detect and report these domain-specific non-motor outcomes.

Existing studies are limited in scope, focusing on a narrow range of non-motor domains and capturing outcomes predominantly in the acute phase (90-day assessments).^10–12^ Crucially, no prior investigations have characterised the full profile of non-motor outcome prevalence across multiple domains or examined whether mRS categories adequately reflect the broader burden of post-stroke disability. Therefore, we aimed to address this gap by systematically evaluating 13 non-motor health domains at 6 months after IVT, EVT, or both, stratifying prevalence by mRS score and identifying baseline predictors associated with adverse non-motor outcomes.

## Methods

### Design, study setting and participants

For this prospective observational cohort study, we used data from the Stroke Investigation Group in North and Central London (SIGNAL) registry, based at the comprehensive stroke unit of University College London Hospitals (UCLH) NHS Foundation Trust. The UCLH stroke unit serves a diverse population of approximately 1.6 million individuals across 5 North Central London boroughs: Camden, Islington, Haringey, Enfield and Barnet. We included patients who received intravenous IVT, EVT or both treatments for acute ischaemic stroke between 1 January 2017 and 13 January 2020.

### Inclusion and exclusions

We included adults over the age of > 18 years with a confirmed clinical diagnosis of acute stroke based on clinical findings and with evidence of acute ischaemia on admission or 24 h neuroimaging with CT or MRI and who underwent revascularisation therapy with IVT, EVT or both. Only patients treated in accordance with the service treatment guidelines were included which allowed for IVT delivery up to 4.5 h and EVT up to 6 h in eligible patients. CT perfusion was not enabled at our site at this time. Our study team validated each diagnosis by cross-referencing the clinical and neuroradiological reports available in the patients’ electronic health records.

We excluded patients with TIA, subarachnoid haemorrhage (SAH), ICH, and patients who did not receive acute revascularisation treatment. We followed the Strengthening the Reporting of Observational Studies in Epidemiology (STROBE) guidelines for reporting observational studies in epidemiology in writing this manuscript.

### Baseline demographic and clinical data

Sociodemographic data (age, sex, ethnic origin, discharge location) and clinical characteristics (cardiovascular risk factors, medication prescriptions, mRS and admission NIHSS) were extracted directly from the SIGNAL database and individually validated against electronic health records (EPIC).

### Non-motor outcome measures

The methods of non-motor outcome data collection have been reported previously.^8^ Briefly, we measured the non-motor outcomes using 3 widely validated patient-reported health outcome measures recommended by the International Consortium for Health Outcomes Measurement (ICHOM).^13^ These included the Patient-Reported Outcomes Measurement Information System (PROMIS-29 version 2.0) scale, covering anxiety, depression, fatigue, sleep disturbance, pain and participation in social roles and activities; the Barthel index scale, evaluating bowel and bladder dysfunction and the Stroke Impact Scale-59 (SIS-59 version 3.0) scale, addressing memory, communication, ADL/instrumental activities of daily living (IADL), mood and social relationships.^14–16^

Patient-Reported Outcomes Measurement Information System-29 consists of 29 items across 7 domains: physical function, anxiety, depression, fatigue, sleep disturbance, participation in social roles and activities and pain (intensity and interference).^14^ Domain scores are standardised on the US general population T-scale (mean score = 50, SD = 10), with higher mean scores indicating worse outcomes.^14^

Stroke Impact Scale-59 comprises 59 items across 8 domains: strength, hand function, mobility, memory, communication, ADL/instrumental ADL, mood and social relationships.^15^ Each domain is scored on a 0–100 scale, with higher scores reflecting better patient-reported outcomes.^15^ We utilised the bowel and bladder domains of the Barthel index scale to capture autonomic function.^16^

We dichotomised non-motor outcomes, defining an adverse outcome using cutoff scores of ≥ 55 in PROMIS-29, > 2 in the Barthel index scale and ≥ 50 in SIS-59. All scores were oriented so that higher scores indicated worse non-motor outcomes.^14–16^

To ensure comprehensive follow-up and reduce bias, we used a range of methods, including outpatient visits, home visits, telephone interviews and postal questionnaires, all conducted by clinically trained practitioners. For patients with communication difficulties, language barriers or severe disability (mRS 4–5), we allowed proxy responders to assist. All non-motor outcomes were self-reported by patients or their proxies, and all outcome measures used were validated for proxy use to ensure reliable data collection across diverse patient groups.

### Statistical analysis

Patient characteristics and non-motor outcomes were summarised using descriptive models including mean ± SD for continuous variables, median—IQR for ordinal variables, and frequencies (*n*, %) for categorical variables. Sociodemographic and clinical characteristics data were compared across 3 groups (IVT, EVT and combination group) using one-way analysis of variance and Kruskal–Wallis *H* test as appropriate. We estimated the prevalence of each non-motor outcome by performing unadjusted proportion analyses for the whole cohort and across individual mRS scores (0–5) separately. We stratified mRS scores into favourable (mRS = 0–2) and unfavourable (mRS = 3–5) functional outcomes. We used Pearson’s chi-squared test to compare the prevalence of non-motor outcomes across the 2 mRS groups and used the Benjamini–Hochberg false discovery rate procedure to guard against potential false-positive discoveries.

We evaluated factors associated with adverse prevalence of non-motor outcomes, using the multivariable logistic regression analysis. The multivariable model included a fixed set of covariates that met predefined significance (*P <* .10) in unadjusted comparisons of sociodemographic and clinical characteristics across groups. These included a previous history of stroke/TIA; stroke recurrence within 6 months; pre-morbid mRS; discharge location; age; sex; ethnic origin and stroke severity on admission as measured by NIHSS. Results of the multivariable analysis were reported as odds ratios and 95% Cl. All statistical analyses were performed on STATA version 18 software.

## Results

### Baseline characteristics

**Figure 1:**
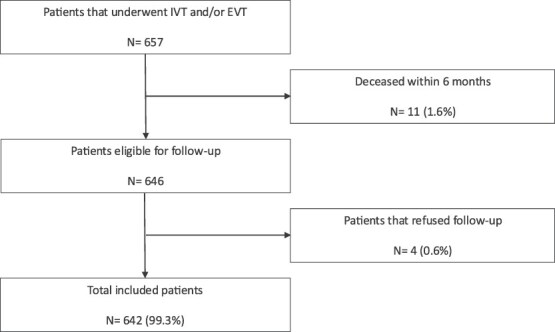
Patient selection. Abbreviations: IVT = intravenous thrombolysis; EVT= Endovascular Thrombectomy.

Between January 2017 and January 2020, a total of 657 patients underwent IVT, EVT or both treatments. Of those, 11 (1.6%) died before the 6-month follow-up, and 4 (0.6%) refused follow-up. Therefore, a total of 642 out of 646 surviving patients (99.3%) who underwent IVT (*n* = 382), EVT (*n* = 153) or both (*n* = 107) for acute ischaemic stroke were included in our analysis (see Figure 1).

The baseline characteristics of the patients are summarised in Table 1. The median age was 73 years (IQR = 62–82), and 279 (43.5%) were female. Among all treatment groups, the median admission NIHSS score was 5 (IQR = 2–9), and the median 6-month mRS was 1 (IQR 0–3). Compared with patients who received IVT alone, those treated with EVT were significantly less likely to have a history of stroke or TIA (14.4% vs 21.0%, *P* = .029) and stroke recurrence at 6 months (5.9% vs 10.2%, *P* = .054). Endovascular thrombectomy patients also had significantly better outcomes on the 6-month mRS (median 1 vs 2, *P* = .034) and were more likely to have received an early supported discharge plan (63.5% vs 57.5%, *P* = .040) compared to the IVT group.

**Table 1:** Baseline characteristics.

Characteristics	All	IVT	EVT	Both	*P*
	642	382	153	107	
Age median, range (IQR)	73 (62–82)	72 (56–78)	72 (60–80)	70 (52–80)	.2914
Female sex *n* (%)	279 (43.5%)	154 (40.3%)	73 (47.7%)	52 (48.6%)	.937
**Ethnicity *n* (%) 627**					
White	375 (59.8%)	216 (58.2%)	91 (59.9%)	68 (65.3%)	.294
Asian	112 (17.4%)	73 (19.6%)	25 (16.5%)	14 (13.1%)	…
Black	85 (13.6%)	55 (14.8%)	17 (11.1%)	13 (12.1%)	…
Other	55 (8.8%)	27 (7.2%)	19 (12.5%)	9 (8.4%)	…
**Medical history *n* (%)**					
Previous stroke/TIA	121 (19.5%)	80 (21%)	22 (14.4%)	19 (17.8%)	**.029**
Stroke reoccurrence	58 (9.1%)	39 (10.2%)	9 (5.9%)	10 (9.3%)	**.054**
Hypertension	406 (63.2%)	243 (63.6%)	100 (65.4%)	63 (58.8%)	.922
Dementia	12 (1.9%)	7 (1.8%)	2 (1.3%)	3 (2.8%)	.813
Heart failure	68 (11%)	41 (11.2%)	16 (10.8%)	11 (10.5%)	.942
Diabetes miletus	160 (25.8%)	93 (25.5%)	43 (28.4%)	24 (22.8%)	.138
AF	152 (23.8%)	88 (23.2%)	37 (24.3%)	27 (25.2%)	.690
Smoking history	175 (28.0%)	110 (29.5%)	40 (26.5%)	25 (25.0%)	.899
**Medication history *n* (%)**					
Anticoagulant	157 (24.5%)	84 (22.0%)	51 (33.3%)	22 (20.5%)	**.013**
Antiplatelet	154 (24.4%)	83 (22.2%)	39 (25.5%)	32 (30.5%)	.821
Statin	267 (43.3%)	154 (41.6%)	71 (48.2%)	42 (42.4%)	.974
Antihypertensive	400 (62.3%)	236 (61.3%)	97 (63.3%)	67 (62.6%)	.795
**Clinical outcomes (median, IQR)**					
Admission NIHSS	5 (2–9)	6 (2–9)	6 (2–9)	7 (2–8)	.7720
Pre-morbid mRS	1 (0–2)	1 (0–2)	2 (1–3)	2 (1–3)	.2870
Discharge mRS	3 (1–4)	2 (1–4)	1 (1–4)	2 (0–5)	.5937
6 months mRS	1 (0–3)	2 (1–5)	1 (0–3)	2 (1–4)	**.034**
**Discharge location *n* (%) 620**					
Home	412 (66.5%)	246 (66.7%)	95 (64.6%)	71 (68.2%)	.968
ASU	159 (25.7%)	97 (26.3%)	41 (27.8%)	21 (20.2%)	…
Care home	49 (7.9%)	26 (7.0%)	11 (7.4%)	12 (11.5%)	…
ESD support	367 (58.7%)	212 (57.5%)	94 (63.5%)	61 (57.5%)	**.040**
Proxy responders	38 (5.9%)	17 (4.4%)	13 (8.4%)	8 (7.4%)	.563

Abbreviations: AF = atrial fibrillation; ASU = acute stroke unit; ESD = early supported discharge; IVT = intravenous thrombolysis; EVT = Endovascular Thrombectomy. Significant associations are shown in bold.

### Non-motor outcomes

**Figure 2:**
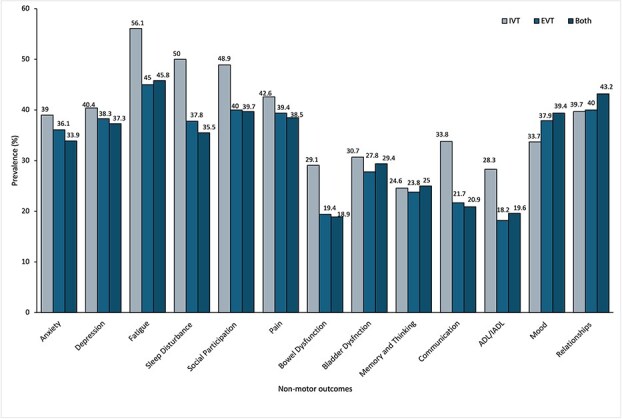
Prevalence of adverse non-motor outcomes by IVT, EVT or both. Estimates for each non-motor outcome domain are reported as proportions (%) of affected survivors. Of the 642 patients included, 382 were treated with intravenous thrombolysis, 153 with endovascular thrombectomy and 107 received both treatments. Across treatment groups, the prevalence of adverse non-motor outcomes ranged from 18% to 56%. Abbreviations: IVT = intravenous thrombolysis; EVT= Endovascular Thrombectomy.

Figure 2 shows the unadjusted prevalence of adverse non-motor outcomes by treatment group (IVT, EVT or both). Among 642 patients treated with IVT, EVT or both, adverse non-motor outcomes were common across all domains, with prevalences ranging from 18% to 56%. In patients treated with IVT alone (*n* = 382), fatigue (56.1%), sleep disturbance (50%), reduced social participation (48.9%) and pain (42.6%) were most prevalent. Among patients treated with EVT (*n* = 153), fatigue (45%), reduced social participation (40%), pain (39.4%) and depression (38.3%) were the most common outcomes.

**Figure 3:**
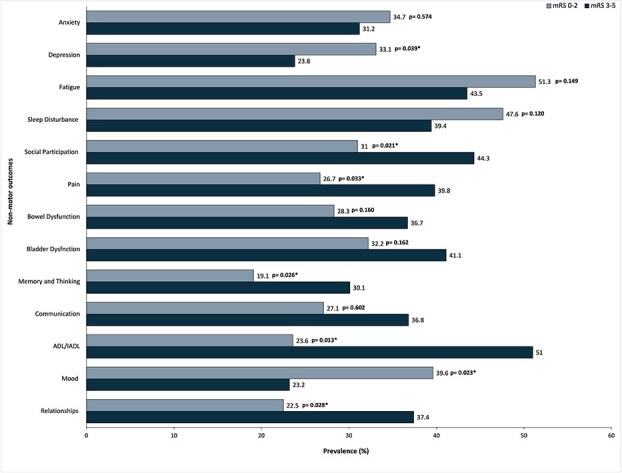
Prevalence of adverse non-motor outcomes in patients with favourable (mRS = 0–2, *n* = 409) vs unfavourable (mRS = 3–5, *n* = 233) 6-month functional outcomes.

Figure 3, Table S1 and Figure S1 show the prevalence of non-motor outcomes stratified by mRS score, with scores of 0–2 indicating a favourable functional outcome and scores of 3–5 indicating an unfavourable outcome. Among patients with favourable mRS scores (*n* = 409), the prevalence of non-motor outcomes ranged from 19% to 51.3%, with fatigue (51.3%), sleep disturbance (47.6%), mood problems (39.6%) and anxiety (34.7%) being most common. Among those with unfavourable mRS scores (*n* = 233), prevalence ranged from 23.2% to 51.0%, with dependency in ADL/IADL (51.0%), reduced social participation (44.3%), fatigue (43.5%) and bladder dysfunction (41.0%) being the most common.

### Adjusted logistic regression analysis

Results from the adjusted multivariable logistic regression analyses are shown in Table 2. Variables that differed significantly between treatment groups at baseline, including previous stroke or TIA, early supported discharge and stroke recurrence within 6 months were included in the adjusted models. Age, sex, admission NIHSS score, ethnic origin and premorbid mRS score were also included a priori on the basis of clinical relevance.

**Table 2:** Baseline factors associated with adverse non-motor outcomes in multivariable adjusted analyses.

	Age ≥ 55 years	Female sex (Ref: Male sex)	Ethnic origin (Ref: White)	Previous stroke or TIA	Stroke reoccurrence	Pre-morbid mRS (Ref: 0–1)	ESD support	Admission NIHSS (Ref: < 5)
**Domains**	**OR (95% Cl) *P* value**							
Anxiety	1.03 (0.74–1.15) *P* = .481	1.08 (0.94–1.11) *P* = .878	0.89 (0.72–1.02) *P* = .563	0.98 (0.67–1.09) *P* = .574	**1.82 (1.04–3.03)** ***P* = 0.048**	1.01 (0.78–1.08) *P* = .977	**1.07 (1.02–1.19)** ***P* = .037**	1.09 (0.85–1.13) *P* = .599
Depression	1.05 (0.93–1.14) *P* = .098	**1.27 (1.11–1.62)** ***P* = .013**	0.97 (0.78–1.07) *P* = .747	1.03 (0.87–1.07) *P* = .926	**2.07 (1.71–3.08)** ***P* = .002**	1.02 (0.87–1.08) *P* = .087	0.79 (0.68–1.02) *P* = .112	**1.54 (1.12–2.08)** ***P* = .003**
Fatigue	**1.34 (1.16–1.63)** ***P* = .001**	1.04 (0.97–1.09) *P* = .832	**2.03 (1.62–3.15)** ***P* < .001**	**1.91 (1.68–3.17)** ***P* < .001**	**1.78 (1.55–2.97)** ***P* < .001**	1.01 (0.81–1.06) *P* = .907	1.07 (0.92–1.11) *P* = .098	**1.57 (1.23–2.31)** ***P* < .001**
Sleep disturbance	**1.16 (1.09–1.32)** ***P* = .003**	0.97 (0.68–1.03) *P* = .086	0.79 (0.65–1.01) *P* = .437	0.87 (0.72–1.01) *P* = .076	1.37 (0.97–1.48) *P* = .516	1.03 (0.95–1.09) *P* = .736	**1.71 (1.52–2.13)** ***P* < .001**	**1.87 (1.25–2.53)** ***P* = .001**
Social participation	0.98 (0.77–1.02) *P* = .344	1.02 (0.95–1.07) *P* = .826	0.95 (0.88–1.02) *P* = .079	**1.51 (1.17–1.82)** ***P* = .001**	**1.50 (1.32–2.07)** ***P* = .003**	1.01 (0.89–1.07) *P* = .523	**1.23 (1.14–1.61)** ***P* = .023**	**1.17 (1.11–1.48)** ***P* = .021**
Pain	**1.23 (1.12–1.56)** ***P* = .010**	**1.36 (1.18–1.61)** ***P* = .001**	0.96 (0.78–1.06) *P* = .072	0.95 (0.82–1.09) *P* = .821	**2.09 (1.81–3.07)** ***P* < .001**	**1.27 (1.13–1.58)** ***P* = .005**	1.02 (0.99–1.07) *P* = .079	0.96 (0.73–1.04) *P* = .181
Bowel dysfunction	**1.98 (1.57–2.30)** ***P* < .001**	0.96 (0.79–1.03) *P* = .093	1.04 (0.97–1.09) *P* = .896	1.03 (0.87–1.06) *P* = .767	0.97 (0.81–1.05) *P* = .305	0.98 (0.86–1.09) *P* = .762	1.03 (0.93–1.11) *P* = .479	**1.41 (1.18–1.56)** ***P* < .001**
Bladder dysfunction	1.02 (0.95–1.08) *P* = .079	**1.08 (1.02–1.14)** ***P* = .029**	0.89 (0.72–1.02) *P* = .297	1.04 (0.95–1.10) *P* = .109	1.25 (0.65–2.29) *P* = .522	**1.32 (1.21–1.44)** ***P* < .001**	**1.27 (1.13–1.70)** ***P* = .004**	**1.37 (1.18–1.59)** ***P* = .002**
Memory and thinking	**1.77 (1.38–2.03)** ***P* < .001**	**1.94 (1.63–2.13)** ***P* < .001**	1.02 (0.94–1.08) *P* = .539	**1.34 (1.11–1.53)** ***P* = .006**	**1.16 (1.09–1.33)** ***P* = .001**	**1.13 (1.07–1.21)** ***P* = .003**	0.94 (0.87–1.03) *P* = .198	**1.07 (1.01–1.12)** ***P* = .017**
Communication problems	1.02 (0.91–1.11) *P* = .866	0.97 (0.82–1.05) *P* = .149	1.02 (0.78–1.08) *P* = .089	**1.54 (1.32–1.79)** ***P* < .001**	**1.98 (1.62–2.37)** ***P* < .001**	1.01 (0.88–1.07) *P* = .173	1.02 (0.90–1.12) *P* = .682	**1.08 (1.01–1.19)** ***P* = .029**
Social relationships	1.00 (0.94–1.09) *P* = .078	0.89 (0.76–1.02) *P* = .102	0.93 (0.78–1.02) *P* = .403	**1.34 (1.11–1.58)** ***P* = .018**	1.01 (0.96–1.09) *P* = .134	**1.27 (1.19–1.37)** ***P* = .003**	**1.18 (1.03–1.41)** ***P* = .048**	**1.58 (1.42–1.83)** ***P* < .001**
ADL/IADL	**1.87 (1.43–3.09)** ***P* < .001**	**1.73 (1.56–2.09)** ***P* < .001**	0.88 (0.74–1.02) *P* = .349	1.02 (0.94–1.06) *P* = .069	**1.32 (1.21–1.74)** ***P* = .003**	**1.57 (1.32–1.84)** ***P* < .001**	**1.56 (1.38–1.76)** ***P* = .002**	**1.92 (1.72–2.30)** ***P* < .001**
Mood problems	0.78 (0.65–1.02) *P* = .059	**1.34 (1.13–1.57)** ***P* < .001**	0.97 (0.84–1.03) *P* = .403	**1.13 (1.02–1.27)** ***P* = .017**	**1.56 (1.29–1.78)** ***P* = .002**	1.02 (0.98–1.07) *P* = .160	1.03 (0.96–1.08) *P* = .499	1.02 (0.87–1.09) *P* = .808

Abbreviations: ADL, activities of daily living; ESD, early supported discharge; IADL, instrumental activities of daily living; OR, odds ratio.

Age was entered as a dichotomous variable (≥55 vs < 55 years). Significant associations are shown in bold.

Multiple multivariate logistic regression analysis identified that stroke recurrence within 6 months, admission NIHSS score > 5, female sex, a history of previous stroke or TIA and age > 55 years were independently associated with 5–10 adverse non-motor outcome domains at 6 months following revascularisation with IVT, EVT or both.

## Discussion

In this observational study of 642 patients treated with IVT, EVT or both for acute ischaemic stroke, we found that adverse non-motor outcomes are prevalent at 6 months post-treatment. Across all treatment modalities, 18%–56% of patients reported significant non-motor symptoms in the various domains. The most frequently reported non-motor outcomes were fatigue, sleep disturbances, reduced social participation and pain. A substantial burden of non-motor outcomes was evident even among patients with favourable functional recovery (mRS = 0–2), with a profile dominated by fatigue, sleep disturbance, mood problems and anxiety symptoms, which are potentially treatable yet often under-recognised in routine follow-up. By contrast, patients with unfavourable outcomes (mRS = 3–5) showed a different non-motor pattern, characterised by dependency in ADL/IADL, reduced social participation, bladder dysfunction and fatigue, reflecting greater care needs, functional dependency and complexity of rehabilitation.

Our findings underscore a broader conceptual issue in stroke outcomes research. Recovery after stroke is multidimensional, and no single instrument can fully capture its impact on patients and their families. The mRS remains the most widely adopted global functional disability measure, underpinning post-stroke clinical decision-making and serving as the primary endpoint in acute stroke trials. By design, the mRS reflects the impact of impairment on functional independence; therefore, higher scores (mRS 3–5) may also reflect certain non-motor domains, such as restricted social participation, dependence in ADL/IADL and disability arising from cognitive impairment, communication problems or, to some extent, continence dysfunction.^11^ Thus, non-motor outcomes are not entirely absent from mRS scores. However, the mRS is not intended to capture multidomain non-motor outcomes. In our study, 19% and 51.3% patients with mRS 0–1, categorised as having no or minimal functional disability, reported fatigue or sleep disturbance, respectively, while 23.2% and 51.0% reported mood problems or anxiety, respectively. These symptoms impose a substantial burden on patients and their families, could also impact daily functioning, yet they frequently remain unrecognised in routine follow-up unless systematically assessed.

Our findings are consistent with emerging post-hoc data from previous studies, which highlight the prevalence of non-motor outcomes following reperfusion therapies in acute ischaemic stroke, even in patients with favourable functional recovery. Much of the existing evidence comes from secondary analyses or supplemental data from phase 3 trials, which typically assess only a limited number of non-motor domains. For example, the REVASCAT trial (*n* = 206) found that 44% of patients reported pain and 36% reported anxiety or depression at 90 days despite a favourable mRS score (0–2).^17^ Similarly, the DEFUSE 3 trial (*n* = 182) identified high rates of fatigue (45%) and depression (32%) at 90 days, even in patients with significant motor recovery. Post-hoc analyses of the Direct MT (*n* = 433), ESCAPE (*n* = 315) and WAKE UP (*n* = 503) trials found anxiety and depression prevalence rates ranging from 38.8% to 43.8%.^10^^,^^18–20^ These results are consistent with our findings, but prior studies were limited by their narrow focus on a few non-motor domains and short follow-up periods. Additionally, many of these studies combined anxiety and depression into a single category, despite the distinct and significant impact these conditions have on stroke survivors. Furthermore, previous studies rarely provided a comprehensive multi-domain profile of non-motor outcomes, particularly those affecting patients with unfavourable outcomes, such as bladder dysfunction and reduced social participation. In contrast, our study assessed 13 non-motor domains individually, providing a more comprehensive and nuanced understanding of the long-term impact of stroke.

Previous studies have identified several key risk factors for adverse non-motor outcomes after stroke, including stroke severity (measured by NIHSS), older age, female sex and a history of prior stroke or TIA. Our study similarly found that higher NIHSS scores on admission, older age, female sex and a history of prior stroke or TIA were significantly associated with non-motor impairments.^10^^,^^11^^,^^18–20^ In addition, we found that stroke recurrence within 6 months was independently associated with adverse non-motor outcomes across multiple domains. These findings align with previous reports indicating that stroke severity and demographic factors are important predictors of long-term non-motor symptoms.^10^^,^^11^^,^^18–20^ However, our study extends these findings by providing a more detailed analysis of non-motor outcomes across a broader range of domains and by demonstrating that these factors are associated with persistent symptoms even in patients with favourable motor recovery (mRS 0–2).

Our findings build on previous reports indicating that non-motor sequelae remain common despite reperfusion therapies, including among individuals classified as having a favourable mRS outcome.^11^ Patients with greater neurological deficit (higher NIHSS), residual disability (higher mRS), recurrent stroke and older age appear particularly vulnerable. Although patient-reported outcome research in stroke is expanding, structured clinical management of these multidimensional impairments remains underdeveloped. Reliance on the mRS alone is insufficient to characterise lived experience after stroke. Our work suggests a need to identify patients at highest risk of adverse non-motor outcomes at discharge to allow mitigating strategies to be implemented.

The proposed framework could include 3 components: (1) risk stratification using routinely collected variables (admission NIHSS, discharge mRS, recurrent stroke, stroke subtype, age and pre-stroke cognitive impairment); (2) tiered screening with universal brief assessments before discharge and at 6–8 weeks, followed by domain-specific predefined referral thresholds and (3) multidisciplinary review, including community-based remote case discussion and reassessment at 6 months standard stroke follow-up clinic.

Our study has limitations. As an observational cohort study, residual confounding may have influenced our results, and patient-reported outcomes could reflect psychosocial factors not captured in our dataset. While our 6-month follow-up provides valuable insights into subacute recovery, long-term outcomes remain underexplored. Moreover, we did not adjust for treatment modality or test its impact on individual non-motor outcome domains, as the primary aim of our study was to assess the overall prevalence of adverse non-motor outcomes in patients who received gold-standard treatments. To validate our findings, identify impact of the treatment modality, and incorporate multi-domain non-motor outcomes into prognostic models and clinical care pathways, larger, more diverse cohorts are needed. Moreover, our data are limited to a single follow-up timepoint at 6 months post-stroke. Future studies should incorporate assessments during the hyperacute and early subacute phases, as well as longer-term follow-up, to better characterise the trajectory and evolution of non-motor outcomes among patients treated with IVT, EVT or combined therapies. However, our study underscores the importance of identifying patients at higher risk for persistent non-motor symptoms. Specifically, patients with higher NIHSS scores, older age, female sex and a history of prior stroke or TIA may benefit from person-centred treatment strategies to address both motor and non-motor recovery.

A major strength of our study is its comprehensive assessment of non-motor outcomes across 13 distinct health domains at 6 months post-treatment. By evaluating a broad range of non-motor symptoms such as fatigue, sleep disturbances, social participation, pain, mood disturbances, memory problems and ADLs we provide a more detailed understanding of the long-term impact of stroke, even in patients with favourable functional recovery (mRS 0–2). This extensive approach allows for a more complete picture of post-stroke recovery after IVT and/or EVT treatments.

## Conclusion

In conclusion, our findings demonstrate that many individuals experience non-motor outcomes after revascularisation treatments with IVT, EVT or both, even among those with favourable mRS scores (0–2). The most affected domains include fatigue, sleep disturbances, reduced social participation, pain and anxiety. Our study highlights the limitations of mRS as the sole outcome measure in stroke recovery, as it fails to capture crucial non-motor outcomes that reflect the true impact of stroke on patients’ lives. These results underscore the need for a more comprehensive approach to stroke recovery, integrating both motor and non-motor outcomes for a to enable more complete understanding of long-term recovery and designing to design person-centred life after stroke pathways.

## Supplementary Material

IVT_EVT_Supplemantery_aakag066

## Data Availability

Fully anonymised data relevant to this study will be shared upon reasonable request from the corresponding author, pending approval by the SIGNAL investigators. A data-sharing agreement must be put in place before any data can be shared.

## Abbreviations

ADL, Activities of daily living; IVT, Intravenous thrombolysis; SIGNAL, Stroke Imaging and Non-motor Outcomes after Acute Ischaemic Stroke; STROBE, Strengthening the Reporting of Observational Studies in Epidemiology; UCLH, University College London Hospitals
