# Supplementary material for: Patient-reported non-motor outcomes after endovascular thrombectomy and intravenous thrombolysis: an observational study
Source: Eur Stroke J. 2026 Jun 22;11(6):aakag066. doi: 10.1093/esj/aakag066 (PMC13286002; doi:10.1093/esj/aakag066)
Supplement: IVT_EVT_Supplemantery_aakag066 [file ivt_evt_supplemantery_aakag066.docx]

**Supplementary File Study Titled: Patient-Reported Non-Motor Outcomes After Endovascular Thrombectomy and Intravenous Thrombolysis: A Large Observational Study**

| Page 2 | Table 1. Prevalence of self-reported health outcomes on physical domains |
| --- | --- |
| Page 3 | Figure 1. Prevalence of adverse non-motor outcomes across mRS scores |

**E-Table 1. Prevalence of non-motor outcomes by mRS scores with unadjusted comparisons and Benjamini-Hochberg false positive rate correction**

| **Non-motor Domain** | **All** | **mRS 0 – 2** | **mRS 3 – 5** | ***P* Value** | **BH *P* Value** |
| --- | --- | --- | --- | --- | --- |
| Anxiety | 39.6% | 34.7% | 31.2% | 0.530 | 0.574 |
| Depression | 38.2% | 33.1% | 23.8% | 0.021 | **0.039*** |
| Fatigue | 53.0% | 51.3% | 43.5% | 0.103 | 0.149 |
| Sleep Disturbance | 46.6% | 47.6% | 39.4% | 0.074 | 0.120 |
| Social Participation | 46.4% | 31.0% | 44.3% | 0.008 | **0.021*** |
| Pain | 41.7% | 26.7% | 39.8% | 0.005 | **0.033*** |
| Bowel Dysfunction | 26.4% | 28.3% | 36.7% | 0.123 | 0.160 |
| Bladder Dysfunction | 35.8% | 32.2% | 41.1% | 0.137 | 0.162 |
| Memory Problems | 24% | 19.1% | 30.1% | 0.006 | **0.026*** |
| Communication | 30.4% | 30.4% | 27.1% | 0.602 | 0.602 |
| ADL/IADL | 25.8% | 23.6% | 51.0% | <0.001 | **0.013*** |
| Mood Problems | 32.9% | 39.6% | 23.2% | 0.007 | **0.023*** |
| Relationships | 33.1% | 22.5% | 37.4% | 0.013 | **0.028*** |

* E-Table 1 shows the prevalence of adverse non-motor outcomes among stroke survivors stratified by functional disability at follow-up (modified Rankin Scale [mRS] 0–2 *vs* mRS 3–5). Percentages represent unadjusted prevalence within each mRS group. Between-group comparisons were performed using unadjusted χ² tests. To account for multiple testing across 13 non-motor domains, p values were corrected using the Benjamini-Hochberg false discovery rate (FDR) procedure. After FDR correction, impairments in activities of daily living/instrumental activities of daily living (ADL/IADL), mood, pain, social participation, memory, and relationships remained significantly more prevalent among patients with greater disability (mRS 3–5), whereas differences in fatigue, sleep disturbance, bowel and bladder dysfunction, anxiety, and communication did not survive multiple testing correction. Positive percentage differences indicate a higher prevalence in the mRS 3–5 group.

**
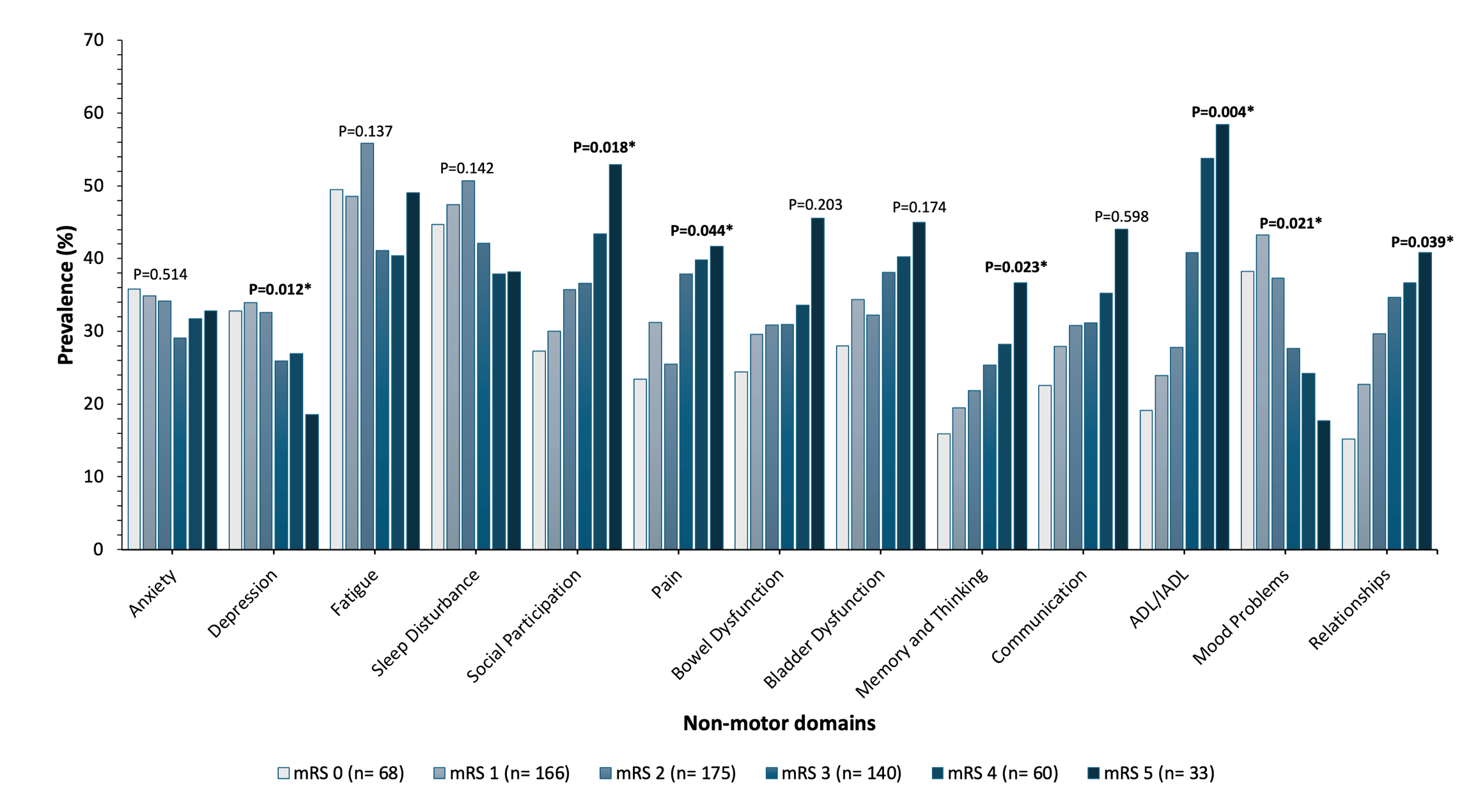
E-Figure 1. Prevalence of adverse non-motor outcomes across mRS scores**
